# Pathogenesis, Diagnosis and Management of Obstetric Antiphospholipid Syndrome: A Comprehensive Review

**DOI:** 10.3390/jcm11030675

**Published:** 2022-01-28

**Authors:** Jaume Alijotas-Reig, Enrique Esteve-Valverde, Ariadna Anunciación-Llunell, Joana Marques-Soares, Josep Pardos-Gea, Francesc Miró-Mur

**Affiliations:** 1 Systemic Autoimmune Diseases Research Unit, Vall d’Hebron Hospital Campus, Vall d’Hebron Institut de Recerca (VHIR), Passeig Vall d’Hebron 119-129, 08035 Barcelona, Spain; ariadna.anunciacion@vhir.org (A.A.-L.); jmarques@vhebron.net (J.M.-S.); jpardos@gmail.com (J.P.-G.); 2 Systemic Autoimmune Diseases Unit, Department of Internal Medicine, Vall d’Hebron Hospital Campus, Hospital Universitari Vall d’Hebron (HUVH), Passeig Vall d’Hebron 119-129, 08035 Barcelona, Spain; 3 Department of Medicine, Faculty of Medicine, Universitat Autònoma de Barcelona (UAB), 08193 Barcelona, Spain; 4 Department of Internal Medicine, Althaia Xarxa Assistencial, Carrer Dr Joan Soler 1-3, 08243 Manresa, Spain; doctor.esteve@gmail.com

**Keywords:** antiphospholipid antibody, non-criteria antiphospholipid antibodies, diagnosis, pathogenesis, management, obstetric antiphospholipid syndrome, review

## Abstract

Antiphospholipid syndrome is an autoimmune disorder characterized by vascular thrombosis and/or pregnancy morbidity associated with persistent antiphospholipid antibody positivity. Cases fulfilling the Sydney criteria for obstetric morbidity with no previous thrombosis are known as obstetric antiphospholipid syndrome (OAPS). OAPS is the most identified cause of recurrent pregnancy loss and late-pregnancy morbidity related to placental injury. Cases with incomplete clinical or laboratory data are classified as obstetric morbidity APS (OMAPS) and non-criteria OAPS (NC-OAPS), respectively. Inflammatory and thrombotic mechanisms are involved in the pathophysiology of OAPS. Trophoblasts, endothelium, platelets and innate immune cells are key cellular players. Complement activation plays a crucial pathogenic role. Secondary placental thrombosis appears by clot formation in response to tissue factor activation. New risk assessment tools could improve the prediction of obstetric complication recurrences or thromboses. The standard-of-care treatment consists of low-dose aspirin and prophylactic low molecular weight heparin. In refractory cases, the addition of hydroxychloroquine, low-dose prednisone or IVIG improve pregnancy outcomes. Statins and eculizumab are currently being tested for treating selected OAPS women. Finally, we revisited recent insights and concerns about the pathophysiology, diagnosis and management of OAPS.

## 1. Introduction

Antiphospholipid syndrome (APS) is a systemic autoimmune disorder characterized by the occurrence of vascular thrombosis—arterial or venous—and/or pregnancy morbidity [1]. These clinical manifestations, according to the Sydney classification criteria, should be associated with the persistent positivity for antiphospholipid antibodies (aPL) [1,2]. When no other underlying autoimmune disease exists, the syndrome is defined as primary APS. Otherwise, when other autoimmune disorders coexist, mainly systemic lupus erythematosus (SLE), it is named secondary or associated APS [3,4]. In its most severe form, a life-threatening and infrequent condition known as catastrophic APS (CAPS), patients develop multiple organ dysfunction caused by a rapid onset of small vessel thromboses [5]. Women that fulfil the Sydney criteria who did not have previous thrombotic events are identified as obstetric antiphospholipid syndrome (OAPS) patients [6,7].

According to the Sydney clinical criteria, pregnancy morbidity includes (i) at least three consecutive miscarriages before week 10 of gestation, (ii) one or more fetal losses (FL) at ≥ 10 gestational weeks and (iii) stillbirth or prematurity due to eclampsia or severe pre-eclampsia (PE) or placental insufficiency before the 34th week of gestation [2]. Nevertheless, as occurs in other autoimmune diseases, many patients fail to completely meet the Sydney clinical criteria. These cases are heterogeneously defined across the literature [8], and in this review, we classified them as aPL-related obstetric morbidity (OMAPS) [7,9,10,11,12] (Table 1).

Antiphospholipid antibodies are a heterogeneous group of immunoglobulins directed against negatively charged phospholipids, cofactors or phospholipid–cofactor complexes that are usually present in monocyte, trophoblast, endothelial and platelet cell membranes [13,14,15]. The laboratory criteria for APS include the detection of lupus anticoagulant (LA), IgG/IgM anticardiolipin antibodies (aCL) with titers >40 GPL or MPL or >99th percentile or IgG/IgM anti-β2glycoprotein-1 (aβ2GPI) antibodies with titers >40 AU or >99th percentile. Positivity for at least one of the mentioned parameters must be detected at least twice 12 weeks apart [2]. Cases with low titers of aPL or only one positive test, and therefore not in agreement with the Sydney recommendations, are classified as non-criteria OAPS (NC-OAPS; see Table 1). These women could present autoantibodies against other phospholipids, such as phosphatidic acid, phosphatidylserine, phosphatidylinositol, phosphatidylglycerol, phosphatidylethanolamine or phosphatidylcholine; cofactors such as prothrombin, annexin A5 or vimentin; IgA aCL or IgA aβ2GPI. All of them are referred to as non-criteria aPL [8,16].

Antiphospholipid antibodies are detected in 1–5% of healthy women of reproductive age [17]. aPL/APS is the most frequently treatable acquired thrombophilia during pregnancy [18], and its prevalence has increased in the last years. In fact, 10–29% of women with poor obstetric outcomes are carriers of aPL [4].

In the last years, the study of the pathogenic mechanisms leading to the different clinical manifestations of APS has increased, and from these studies, a two-hit hypothesis to explain the development of APS manifestations has been proposed. The presence of aPL is considered the “first hit” that results in the activation of inflammatory—mainly complement-mediated—and coagulation pathways when it is accompanied by another procoagulant trigger, the “second hit” [15,19]. The expression of a tissue factor (TF) in endothelial and innate immune cells, the activation of nuclear factor κB (NF-κB), the release of proinflammatory cytokines, platelet activation, the production of intracellular reactive oxygen species (ROS) and the induction of the complement cascade are some of the most studied molecular events that explain the development of this syndrome [19,20,21].

The accurate diagnosis of OAPS is a prerequisite to optimize clinical management and to avoid poor obstetric outcomes, such as fetal growth restriction (FGR) and/or early onset PE. When the standard of care is used, the rate of pregnancy success can rise up to 80% of cases [4,18,20,22,23,24]. On the other hand, the significant psychological sequelae that may affect the couple, as well as the feeling of blame related to recurrent pregnancy losses and the fear to have more losses, should be considered.

Moreover, these recurrent poor obstetric outcomes suppose an important economic burden for both patients and health systems. The aim of this article is to thoroughly review the concepts related to the pathogenesis, diagnostic, clinical concerns and management of OAPS, as well as to update new data on them.

## 2. Pathophysiology of OAPS

**Figure 1:**
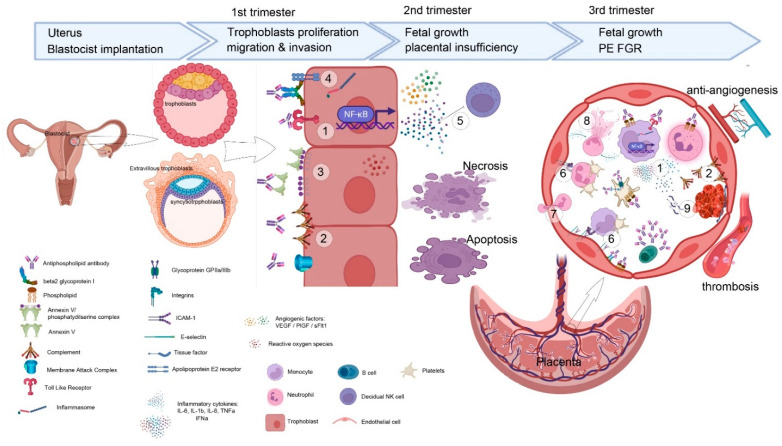
Cellular and molecular mechanisms of action of aPL in the pathophysiology of OAPS. aPL affect different cellular processes from blastocyst implantation in the uterus mucosa to trophoblast proliferation and differentiation and, eventually, the impairment of fetal growth due to antiangiogenic and prothrombotic activation. aPL induces inflammation via TLR4/MyD88 (1) in trophoblasts and endothelium and immune cells. Complement component deposition (2) on the endothelium and on trophoblasts drives inflammation and MAC formation, leading to cell death. Trophoblasts apoptosis is also produced when aPT antibodies expose PS on the external trophoblast cellular membrane (3). Inactivation of eNOS and dysfunction in ROS production are observed when the ApoE2 receptor binds to the aβGPI–βGPI complex (4). Trophoblast perturbation also affects decidual NK activity, crucial for embryo implantation (5). aPL in the lumen vessels of placental arteries and veins has pleiotropic actions: induction of leukocyte adhesion (6) on inflamed vasculature that drives neutrophil infiltration (7) inside the decidua and NET formation in response to ROS production (8). These mechanisms altogether enhance thromboinflammation with activation of the coagulation cascade initiated by TF (9). Legend depicts cells and molecules. Image created in BioRender.com (accessed on 13 December 2021).

Histological studies found that placental infarction, impaired spiral artery remodeling, decidua inflammation and the deposition of complement split products were the most common features in the placentas of aPL-positive women (Table 2). These pathological manifestations suggest the role of thrombotic, antiangiogenic and inflammatory factors in the pathological process of the disease [25,26,27]. aPL have been shown to have a direct embryotoxic effect by affecting both the embryo and the uterus, resulting in decreased implantation or embryo growth [28,29]. aPL display this embryotoxicity by acting on different cellular targets: innate immune cells (neutrophils, monocytes and platelets); endothelial cells and trophoblast cells (Figure 1). The clinical manifestations of OAPS, such as miscarriage, fetal loss, PE or placental insufficiency, could be in response to the action of aPL to any one of these cells. The effect of aPL on immune or endothelial cells leads to blood clotting and decreased angiogenesis. aPL disrupt trophoblasts cell biology with affectation from implantation to the remodeling of spiral arteries [30,31].

Whether aPL from obstetric or thrombotic APS patients elicits different mechanisms of action is unresolved. Only APS patients with thrombotic manifestations carry an increased risk of subclinical atherosclerosis, while patients with OAPS do not, thus leading to the idea that distinctive pathogenic mechanisms might sustain the two conditions [39]. In general, the major mechanism of hypercoagulability in the APS is the aPL-mediated upregulation of TF via the activation of the TLR and NF-κB pathways. These different pathogenic actions were investigated in two in vitro studies. In the first study, immunoglobulins from APS patients with vascular morbidity or from obstetric morbidity differently activated these monocyte signaling pathways [40]. aPL against β2GPI from thrombotic APS patients were the only ones to induce TF production by monocytes [40]. In the second study, purified IgG from patients with obstetric but not IgG from non-obstetric APS inhibited trophoblast invasion [41]. Evidence on the different genetic risk factors for the two APS types has also been investigated [42].

### 2.1. aPL Affects Implantation and Trophoblasts Growth

One of the key early events in the establishment of pregnancy is the development of trophoblast subpopulations from the trophectoderm of the implanting blastocyst. After implantation, the trophectoderm of the blastocyst rapidly proliferates and generates the trophoblast, the unique cell type of the placenta. The development and function of the placenta is supported by the maternal decidua, the uterine mucosa [43]. Trophoblasts differentiate to either extravillous trophoblast cells (EVT) or syncytiotrophoblasts. EVT participate in the remodeling of the uterine spiral arteries that supply the placenta in addition to anchor the placenta to the maternal decidua. Reduced trophoblast invasion and spiral artery transformation are associated with obstetrical disorders such as miscarriages and PE displayed by women with aPL. For their physiological development, trophoblasts require the secretion of angiogenic factors. In in vitro studies, it has been shown that aβ2GPI autoantibodies perturbed the production of vascular endothelial growth factor (VEGF) and placental growth factor (PlGF) [44] while clearly inhibiting the production of VEGF by endometrial cells [45,46]. aβ2GPI autoantibodies also prevent EVT function [47]. aPL antibodies may also affect the syncytiotrophoblast after being internalized by members of the low-density lipoprotein receptor (LDLR) family [48]. Unlike the syncytiotrophoblast, aPL were not internalized by EVT. It would indicate that the aPL mechanisms of action in EVT are different from that in the syncytiotrophoblast, and it would suggest that aPL trigger intracellular signaling cascades through EVT surface receptor interactions [49]. At the cell surface of human first-trimester trophoblasts, aβ2GPI autoantibodies activate Toll-like receptor (TLR)-4/Myd88, and the signaling is transduced to the Nalp3 inflammasome, leading to a proinflammatory state by the production of interleukin (IL)-1β and IL-8 [50,51]. A second intracellular mechanism implies the upregulation of miR-146a-3p, leading to the activation of TLR-8 and the secretion of IL-8 [52]. In addition, the mechanisms of the inflammatory response in trophoblasts induced by aβ2GPI autoantibodies involve the impairment of negative regulators of TLR signaling and autophagy [53]. In turn, autophagy suppression of trophoblasts cells activates decidual NK (dNK) cytotoxicity and inhibits trophoblasts invasion [54]. In the first-trimester, human decidual leukocytes are primary dNK cells [55]. A dysregulated dNK activation during this decidual vasculature remodeling is associated with PE in humans [56].

On the other hand, a clinical study suggested that statins may improve pregnancy outcomes in refractory OAPS by increasing the placental blood flow [57]. The proposed mechanism was that statins increased the endothelial nitric oxide synthetase (eNOS) activity, raising the serum nitric oxide (NO) levels [58]. These studies demonstrated a placental vasculoprotective role for eNOS/NO. The pathogenicity of aβ2GPI autoantibodies was observed in a murine model, where autoantibodies against domain I of β2GPI decreased the bioavailable NO by antagonizing the activity of eNOS. The dimerization of β2GPI by the autoantibodies enables its binding to ApoE receptor 2, whose activation triggers the inhibition of eNOS activity [59]. Thus, aPL-induced leukocyte–endothelial cell adhesion and thrombosis are caused by eNOS antagonism.

### 2.2. aPL Activates Complement in the Pathophysiology of OAPS

The placenta from OAPS women exhibited high levels of C4a and C3b deposition, which indicated the possibility of complement activation in the pathogenesis of pregnancy complications in OAPS women [36]. Some clinical studies have found low plasma C3 and C4 complement levels as independent predictors of lower neonatal birth weight and premature delivery [60,61], and higher serum C3 levels in the first gestational trimester was a protective factor for fetal loss [62]. The PROMISSE study showed that Bb and C5b-9 were significantly higher in patients with poor obstetric outcomes, as indicative of the participation of complement activation in obstetric complications [63]. Studies on murine models have shown that complement activation is essential and causative in aPL antibody-induced fetal injury [64]. Deposition of the human aPL IgG in the mouse’s decidua with subsequent complement activation, neutrophil infiltration and local TNF secretion caused fetal loss and growth retardation [64,65,66]. Complement activation by aβ2GPI autoantibodies induced thrombus formation subsequent to a priming inflammatory factor [67] and required the activation of C3 and C5 [68]. C5 and its cleavage product, anaphylotoxin C5a, are pivotal members of complement activation by aPL. Mice lacking C5 or those treated with an anti-C5 mAb showed less neutrophil infiltration into the decidua and were protected from aPL-induced pregnancy complications [64]. C5a is a potent chemotactic factor and activator of neutrophils. In mice depleted of neutrophils, treatment with aPL did not cause pregnancy loss or growth restriction, nor were inflammatory infiltrates within the decidua. Furthermore, without neutrophil infiltration, there is less C3 deposition [64]. The ultimate action of aPL activation of a complement is the generation of the C5b-9 membrane attack complex (MAC). The deposition of MAC on the endothelium or on trophoblasts leads to endothelial injury and enhances trophoblast apoptosis [69].

Moreover, in response to aPL-generated C5a, neutrophils, monocytes, platelets and endothelial cells express TF potentiating inflammation in the decidua, leading to miscarriages [70]. TF is the major cellular initiator of the coagulation protease cascade and plays important roles in both thrombosis and inflammation. The coagulation cascade is initiated by the complex of TF with factor VIIa (FVIIa). The TF–FVIIa complex activates its substrates: factor X and factor IX. Activated FX (FXa) then converts prothrombin to thrombin, which cleaves fibrinogen and activates platelets, leading to the formation of thrombi. TF also mediates aPL-induced trophoblast injury by two pathogenic mechanisms: (i) complexes of TF with FVIIa and/or FVIIa-FXa induce the production of TNF-α, interleukins and adhesion molecules by cleaving protease-activated receptors (PARs) [71], and (ii) TF generated by neutrophils contributes to respiratory burst with the generation of ROS [72]. Both mechanisms lead to fetal death in experimental models of APS.

### 2.3. Thrombosis versus Complementopathy

Most of the clinical and pathological findings associated with aPL/OAPS seem to have an underlying inflammatory basis more than a primary thrombotic cause [26]. The underlying mechanisms of complement-mediated thrombosis are not fully clarified, but thromboinflammation is a prominent clinical feature of complement activation [73]. Although OAPS is generally treated with anticoagulation therapy, heparin also protects pregnant APS patients from complications through inhibition of the complement [74]. Anticoagulants fondaparinux, a specific inhibitor of FXa, or hirudin, a direct inhibitor of thrombin, did not inhibit the generation of complement split products or prevent pregnancy loss, demonstrating that anticoagulation therapy is insufficient protection against OAPS [74]. Heparin has long been known to possess anticomplement activity. From early studies in 1929, we have identified several mechanisms for this effect, including the inhibition of C1q binding to immune complexes, interference with the interactions of C4 with C1s and C2, blockade of the formation of the C3 amplification convertase by the alternative pathway and inhibition of the formation of the MAC. Girardi’s data [74] indicated that heparins prevent obstetrical complications in women with APS, because they block activation of the complement induced by aPL antibodies targeted to decidual tissues rather than by their anticoagulant effects.

### 2.4. Different aPL with Different Mechanisms of Action

The anionic phospholipid phosphatidylserine (PS) is a procoagulant factor when exposed on the external face of cellular membranes. Annexin A5 binds to PS and forms a protecting anticoagulant complex. However, OAPS patients show reduced levels of Annexin A5 on placental villi, because aβ2GPI autoantibodies disrupt PS–Annexin A5 complexes and promote a thrombogenic state [75]. The ability of β2GPI immune complexes to disrupt the anticoagulant complex is inhibited by hydroxychloroquine (HCQ) [76], which reverses the thrombogenic properties of aPL [77].

As it is described above for eNOS inactivation, the dimerization of β2GPI by aβ2GPI autoantibodies enables its binding to glycoprotein Ibα and ApoE receptor 2 on platelets and potentiates platelets activation [78] and triggers thrombosis in a murine model of APS [79]. The effect of aβ2GPI antibodies on platelet aggregation and ATP release involve heparinase activation [80]. Heparinase inhibition reduces aβ2GPI-dependent platelet activation [80]. In APS patients, activated platelets release microparticles that contribute to recurrent miscarriages by affecting trophoblasts and endothelial cells [81], although the exact mechanism is unclear.

## 3. OAPS: Clinical Manifestations and Classification Criteria

Even though the Sydney classification criteria include a list of poor obstetric outcomes, the diagnosis of OAPS is still a challenge for physicians who now recognize an expanding range of clinical manifestations not included in the current classification criteria (see OMAPS in Table 1). Similarly, there are cases fulfilling only clinical criteria, but they do not reach the laboratory values defining OAPS (see NC-OAPS in Table 1). It can be explained by the fact that the original aim of the international consensus (Sapporo & Sydney criteria) was to standardize multicenter and clinical trials on APS and was not intended to be used for diagnostic purposes in routine clinical practice. However, clinicians use these criteria for diagnosis purposes.

### 3.1. Clinical Phenotypes of OAPS

OAPS include diverse clinical manifestations that can vary over time; some patients repeatedly suffer the same adverse outcomes, and others have different complications. In the clinical setting, OAPS patients may present two main phenotypes: those women with only pregnancy losses and those women with clinical manifestations related to placental insufficiency. A combination of both could also be possible.

#### 3.1.1. Recurrent Miscarriage and Fetal Loss

When other common causes related to recurrent miscarriages (RM) and FL, such as alterations of the embryonic karyotype, maternal endocrinopathies, infectious disorders, congenital or acquired uterine malformations and inherited thrombophilias, have been ruled out, then aPL/OAPS is the major acquired cause for RM and FL [4,18]. RM is one of the most representative obstetric complications related to recurrent aPL positivity [5,9,18]. aPL explains 5–20% of RM [82,83,84,85,86,87,88]. The lack of a clear definition of miscarriage could be the cause of this wide range of prevalence. Along these lines, Cervera et al. [1] studied a cohort of 590 OAPS with 1580 pregnancies and observed that the prevalence of early RM and late FL was 35.4% and 16.9%, respectively. Similarly, Alijotas-Reig et al. [18] reported that, in a cohort of 1000 patients with OAPS, the most frequent poor obstetric outcome was RM (38.6%), followed by FL (25.3%) and fetal deaths (23%). This series showed similar laboratory categories among these poor obstetric outcomes, LA positivity being the most frequent aPL marker. However, given the prevalence of aPL among the healthy population and the high percentage of miscarriages due to embryonic chromosomal alterations, proportionally more frequent at older maternal ages, some experts recommend more studies providing new data to determine the exact relationship between RM and aPL [89].

#### 3.1.2. Stillbirths

aPL/OAPS is strongly related to stillbirths [90,91,92,93,94]. A case–control study of 100 women that experienced a stillbirth after 22 weeks of gestation established a relative risk of four to five-fold in women persistently positive for LA but not in those with a single positivity for aCL or aβ2GPI through the ELISA method [91]. One of the best multicenter series on fetal death and aPL came from the Spanish Network of Collaborative Death Research, showing that positivity for aCL or aβ2GPI was detected in almost 10% of fetal deaths at 20 weeks of gestation. After excluding cases related to other explainable causes, positive cases for IgG and IgM aCL antibodies were associated with five and two-fold more likely fetal deaths, respectively, while IgG aβGPI antibodies were associated with a three-fold increased chance of stillbirth. Therefore, most isotypes were related to an increased relative risk [94].

Treatment administration at the time of OAPS diagnosis can explain the differences between the obstetric complications suffered by these women and those with a history of OAPS that were not diagnosed and, in consequence, not treated. Although it is accepted that RM is the first adverse outcome in many patients with OAPS, it is not uncommon to observe women with one live child with a history of prematurity or low weight who subsequently present RM or recurrent FL. It can be due to a different aPL pathogenicity or a hypothetical embryonic antigenic sensitization by the maternal immune system with a positivity for aPL in subsequent pregnancies [6].

#### 3.1.3. Placental Insufficiency: Prematurity or Stillbirth Related to Early PE and/or Fetal Growth Retardation

The Sydney classification criteria consider severe prematurity (delivery before 34 weeks of gestation) due to PE and/or FGR as the main manifestations of placental insufficiency [2]. This vascular placental injury is measured by (i) the presence of abnormal or uncertain fetal well-being tests (e.g., absence of activity in the antenatal heart rate monitoring test, suggestive of fetal hypoxia), (ii) the study of abnormal fetal-placental Doppler suggestive of fetal hypoxemia (e.g., absence of telediastolic flow in the umbilical artery) or (iii) the diagnosis of oligohydramnios with an Amniotic Fluid Index (AFI) less than 5 or vertical maximum column less than 2 in the setting of an estimated fetal weight less than the 10th percentile for gestational age [95].

PE is a multifactorial and complex placental-mediated disease with late maternal cardiovascular repercussion that shows two main phenotypes defined by specific pathophysiologic mechanisms and, especially, by the time of debut and severity [96,97]. In brief, early PE (<34 weeks) is characterized by chorionic and placental hypoxia generated by abnormal remodeling of the uterine arteries at the time of decidua invasion by the trophoblast before 16 weeks of gestation. This hypoxia is perpetuated by a fetal placental antiangiogenic environment due to the genesis of hypoxia-dependent antiangiogenic factors. It also affects fetal tissues, generating a restriction of their growth, and finally, also provokes maternal multiorgan involvement, giving rise to PE syndrome. On the other hand, the onset of clinical manifestations in late PE due to endothelial dysfunction begins beyond 34 weeks, usually with a minor involvement of fetal tissues and, therefore, with less impact on fetal growth but with non-negligible potential multiorgan dysfunction in the pregnant woman [98,99,100].

It is well-known that aPL are potential inducers of the peri-implantation inflammatory environment that can lead to an abnormal invasion of the trophoblast in the decidua, thus promoting a hypoxic and antiangiogenic “milieu” suitable for inducing the manifestations of placental dysfunction, early PE (<34 weeks) and/or FGR. The relative risk of presenting a placental dysfunction disease in OAPS has been studied, and it ranges from two to four-fold [101]. Cervera et al. (1) in their cohort of 590 OAPS with 1580 pregnancies reported that the prevalence of early PE, eclampsia and placental abruption were 9.5%, 4.4% and 2%, respectively. Similarly, Alijotas-Reig et al. [18] reported 18% early and severe PE. Two prospective and observational studies of women with well-characterized OAPS with RM or FL reported that 9% and 10% of women, respectively, developed severe PE in further pregnancies despite receiving the standard of care (SC) [84,102].

The prevalence of aPL in PE/eclampsia, however, is not yet well-determined. The initial studies reported a prevalence of 14% in cases of severe PE and 28% in HELLP syndrome, a type of severe PE accompanied by non-immune hemolysis, elevated liver enzymes and thrombocytopenia [103]. HELLP syndrome complicates 0.01 to 0.2% of pregnancies in healthy pregnant women and up to 10–12% in patients with OAPS [101,104]. Ferrer-Oliveras et al. [103] analyzed multiple aPL and reported a prevalence of 14.14% in women with PE vs. 7% in the controls, and when atypical aPL were excluded from the analysis, these percentages were 13.19% vs. 3.61%. Concretely, IgM aCL and aß2GPI showed an association with severe and early-onset PE. However, a recent prospective case–control study of unselected women who gave birth due to severe PE or placental insufficiency reported an unexpected result, since only 0.10% of the cases were positive for aPL antibodies compared to 2% of the controls [105]. A rationale for these numbers has not yet been explained, and they should be taken with caution.

The variability in the data reported regarding the association between aPL and placental insufficiency-related poor obstetric outcomes appears to be due to the possible criteria differences in PE or FGR diagnosis and also because of the methods used in the aPL analyses. For the diagnosis of OAPS, some experts argue that second- and third-trimester complications (i.e., unexplained stillbirth or preterm birth from placental insufficiency) are more specific than RM, since the latter can be explained by many other causes different than aPL positivity. The lack of solid data suggests that larger studies with well-defined clinical criteria and with centralized and standardized laboratory determinations are needed to improve and redefine OAPS diagnosis considering all the clinical manifestations related to this syndrome.

## 4. OMAPS and NC-OAPS: Diagnostic Concerns

As previously commented, in a daily clinical setting, there are women that seek medical attention for obstetric complications that could be related to aPL but without fulfilling the full-blown Sydney criteria. Two different situations can be considered: (i) clinical but no laboratory criteria are present and (ii) laboratory but no clinical criteria are present. It poses a challenge for physicians and supposes extra stress for patients.

### 4.1. NC-OAPS

There are women fulfilling only the clinical criteria but failing to reach the laboratory values defining OAPS [7,9]. There are cases with low aCL and/or aβ2GPI titers, only one positive test for LA and/or aCL and/or aβ2GPI or only showing positivity for non-criteria antiphospholipid antibodies e.g., IgG or IgM or IgA isotypes of anti-phosphatidylserine/prothrombin (aPS/aPT), anti-phosphatidylethanolamine (aPE), anti-annexin A5 (aAnA5) and even anti-aβ2GPI-domain 1 [8,10,11,12,13,106,107,108]. These cases are known as seronegative or non-criteria APS [109,110]. Thus_,_ Litvinova et al. [111] showed that, in patients with a clinical picture highly suggestive of OAPS but who persistently tested negative for classical aPL, up to 40% tested positive for at least one non-criteria aPL, while some tested positive for more than one.

Other scenarios should also be considered to explain this issue: (i) antiphospholipid antibody variability due to treatment administration. Variations in the aPL profile; titers and isotypes in response to patient’s treatment, e.g., patients on heparin, prednisone or HCQ, have been reported. This scenario has encouraged some authors to request a redefinition of the OAPS criteria: (ii) pregnancy and aPL variability. The fact that pregnancy may induce a fluctuation [112], reduction or fall in aPL titers in both treated and nontreated women [113,114] should also be considered. This situation may be more frequent in cases treated with low-dose prednisone [115], HCQ or heparin [116,117,118] (Table 3). Thus, a negative aPL test during pregnancy, particularly if heparin is administered, should not rule out the diagnosis of OAPS; in such cases, other tests for classical and noncriteria aPL should be studied for implementation in the future. Finally, it has been reported that up to 50% of women with OAPS may have lower aCL and/or aβ2GPI in the absence of LA compared to those with thrombotic APS [119]. Similar results were reported by Gardiner et al. [120]. At least two different studies from the EUROAPS study group, referring to the outcomes between groups and criteria and non-criteria laboratory OAPS, showed the same obstetric events in cases treated or non-treated with SC [120,121]. (iii) Laboratory pitfalls or technical limitations in ELISA tests [13,122]. In fact, disparity in the results for aPL tested by ELISA, either commercial kits or “homemade” methods that lead to high interlaboratory, intra-laboratory or inter-method variability, has been recognized. In addition, the results of aPL tested by ELISA are reported in arbitrary units based on the calibrator standards. Only aCL IgG/IgM are measured using a unique internationally accepted calibrator (Harris standards). To improve these troubles, additional technologies to detect aPL, such as immunodotblot and thin-layer chromatography with immunostaining, have been developed [111,123], with apparently good results. Ortona et al. [124] and Conti et al. [123] reported 55% and 60%, respectively, of repeated positivity for aPL, tested with non-standardized technologies, in patients having clinical features suggesting APS but with negative aPL results obtained by ELISA. (iv) Lupus anticoagulant test variabilities. Disparity in LA tests exist despite the recommendations of The International Society on Thrombosis and Haemostasis (ISTH) [125] or The British Committee for Standards in Haematology (BCSH) [126]. False-positive LA can be due to contamination of the sample with inhibitor drugs like heparin. More controversial is the risk conveyed by isolated and weak LA. Up to 50% of cases with weak LA values detected in patients with thrombotic APS and OAPS had become negative by the second test [127]. By contrast, false-negative LA tests due to laboratory pitfalls may potentially raise the likelihood of recurrent thrombotic or obstetric events [127]; last, (v) the non-acceptance of non-criteria aPL or isotypes as diagnostic markers. The role played by IgA isotypes is controversial. Although some authors have reported that IgA aCL and aβ2GPI may show positivity in seronegative IgG/IgM [128], others did not find IgA aPL useful for OAPS diagnosis, particularly due to the lack of standardization of the assay methods used [129]. Similar concerns happen with other non-criteria aPL, mainly aPS/PT antibodies. Once again, the value of these aPL is disputed. A systematic review on this topic reported an increased risk for thrombosis, not for obstetric morbidities, in patients who tested positive for aPS/PT antibodies [130], while other authors reported a relationship between aPS/PT and obstetric morbidity [131].

To conclude, there is a rising concern in clinical practice on how to classify and treat these women.

### 4.2. OMAPS

There are women with a history of repeated poor obstetric outcomes who do not fulfil the Sydney criteria for obstetric morbidity but meet the laboratory criteria for OAPS. No other etiology to explain these pregnancy troubles exists. In one study from EUROAPS, clinical features, laboratory data and fetal–maternal outcomes were compared between 1000 women with OAPS and 650 OMAPS. Although several differences were observed between the laboratory categories and obstetric morbidity, the fetal–maternal outcomes were similar in both groups when treated [7]. With this data, the question is whether having two miscarriages of normal embryos at 9 weeks of gestation is really so different from suffering one fetal loss at 10 weeks. Same rationale can be applied for women being diagnosed with PE at 34 weeks of gestation or at 34 + 4 weeks or even when PE is diagnosed before week 34 of gestation but the delivery is after week 34. Similar concerns about the classification and management of these cases have been discussed by some experts in APS in international meetings, highlighting the differences between items included in the Sydney criteria and the facts found in daily clinical practice [8,11,132].

## 5. Catastrophic Antiphospholipid Syndrome

Catastrophic antiphospholipid syndrome (CAPS), also known as Asherson’s syndrome, is a life-threatening clinical form of APS that affects less than 1% of APS cases [133]. In almost 50% of cases, CAPS may be the debut of APS [134]. The main pathophysiologic mechanism related to CAPS is the activation of the classic complement pathway with proinflammatory cytokine release. Ischemia/infarcts of the kidneys, heart, bowels, lungs or brain are most frequent, followed by adrenal, testicular, splenic, pancreatic or skin involvement [135]. The occlusion of small vessels is characteristic, resulting in symptoms related to dysfunction of the affected organs. Depending on the involved organs, patients might have hypertension and renal impairment, acute respiratory distress syndrome (ARDS), alveolar hemorrhage and capillaritis, confusion and disorientation, abdominal pain and distention secondary to bowel infarction or multiorgan failure [134,135].

In brief, the definite CAPS classification criteria include (i) the involvement of three or more organs, systems and/or tissues; (ii) the development of manifestations simultaneously or in less than one week; (iii) confirmation by the histopathology of small vessel occlusion in at least one organ or tissue and (iv) laboratory confirmation of the presence of aPL [134]. When a patient meets all four criteria but it involves only two organs, systems and/or tissues; the absence of laboratory confirmation owing to the early death of a patient never tested for aPL; no confirmation by histopathology analyses or when the development of a third event occurs between one week and one month after presentation, despite anticoagulation, then the patient is classified as probable CAPS [134,136]. Clinicians should keep on high alert for this rapidly aggressive syndrome to perform an early diagnosis and aggressive treatment. In aPL-positive patients and in those having APS, CAPS can be induced by different triggers, such as surgical procedures; severe trauma; infections; malignancies; disseminated intravascular coagulation; drugs, i.e., thiazides, captopril, cyclosporine; anticoagulation withdrawal and even rivaroxaban, SLE flare or pregnancy [134,137]. CAPS overlaps the clinical and laboratory features of thrombotic microangiopathies, such as thrombotic thrombocytopenic purpura, hemolytic uremic syndrome, severe sepsis and type II thrombocytopenia thrombosis induced by heparin or HELLP syndrome. Clinicians must be especially cautious in cases of pregnant women with clinical and laboratory features of HELLP syndrome or thrombotic microangiopathy, although no previous data on aPL status are known. Whether prompt aggressive treatment is started, the overall mortality can be reduced to 30–35% [5,138]. Treatments include the rapid administration of intravenous heparin, corticosteroids, plasma exchange and/or intravenous immunoglobulins or rituximab [138]. Cyclophosphamide is recommended in cases of secondary APS associated with SLE.

Although eculizumab have been kept for refractory cases [138,139], preliminary clinical evidences suggest using it as a first-line therapy.

## 6. Risk Profiles and Risk Scores in OAPS Patients

Despite the numerous efforts made by the scientific community to establish the risk of new obstetric complications using profiles for aPL, such associations are still poorly established today [6,140,141]. Until now, four scores (Global APS score, GAPSS; adjusted GAPSS, aGAPSS; aPL-score, aPL-S and the risk scale of APS) have been developed to improve the risk assessment of suffering thrombosis or obstetric adverse events in patients with aPL, even though they have not been diagnosed with APS [142,143,144,145,146,147]. Approximately 20% of primary OAPS patients, even those under the SC treatment of low-dose aspirin (LDA) combined with low molecular weight heparin (LMWH) during pregnancy, are at a risk of suffering from obstetric morbidity (refractory OAPS) [6,148]. Primary OAPS is also associated with an increased risk of thrombosis [149,150,151,152]. Thus, stratifying these patients according to their risk of developing any clinical manifestations of OAPS could be helpful to select those who might benefit from receiving additional treatment. Different attempts to sort out this issue have been proposed. The European League Against Rheumatology, EULAR, defines aPL profiles as low- or high-risk [22]: (i) high-risk: the presence of LA in ≥two occasions at least 12 weeks apart or triple or double positivity (any combination of LA, aCL and aβ2GPI) or the presence of persistently high aPL titers and (ii) low-risk profile: positivity for aCL or aβ2GPI at low-medium titers, particularly if transiently positive. This categorization has not been thought to assess pure OAPS. In this case, LA and triple positivity have been described as the highest-risk aPL profiles and the aCL IgG isotype persistently positive at high titers as the most frequent one [153]. Recently, Sciascia et al. [143,147] proposed a risk score to stratify APS patients according to their risk of developing thrombotic manifestations, the Global APS Score (GAPSS) and its subsequent adjusted version (aGAPSS) (Table 4). Currently, there is no clear aGAPSS cut-off value assessing this thrombotic risk. The APS ACTION team [154] reviewed and calculated the cumulative aGAPSS of an OAPS cohort. They found the mean aGAPSS value of 11.5 significant to detect patients with higher possibilities of developing obstetric morbidity supposedly related to placental thrombotic events. Udry et al. [153] reported that, in women with OAPS, an aGAPSS ≥7 is an independent risk factor associated with a first thrombotic event. Data from the EUROAPS study group concurred with this result, also proposing aGAPSS ≥ 7 as the cut-off value for thrombotic risk (Morales-Perez et al., underreported data). Recently, Pregnolato et al. [155] implemented a novel algorithm known as EUREKA that considered patients fulfilling the laboratory Sydney criteria and also patients with titers lower than the thresholds accepted as classification criteria. This algorithm refines the current process of risk stratification, taking into account aPL tests routinely performed in every laboratory. It allows clinicians to immediately visualize the risk for the specific aPL profile. Studies are ongoing to better address this topic. The EULAR expert committee is also currently working in this field.

## 7. Clinical Management

The management of women with OAPS includes a close surveillance and tailored treatment before, during and after pregnancy to optimize the maternal and fetal outcomes. A well-balanced team that includes obstetricians and internists, rheumatologists or immunologists with contrasted expertise in this field is mandatory.

### 7.1. Preconception Counselling

As stated in the EULAR recommendations and others [4,6,22], it is fundamental to know any previous pregnancy complication and/or thrombotic event; the presence of the so-called non-criteria manifestations; other associated autoimmune diseases, mainly SLE or Sjögren syndrome; possible genetic thrombophilia; major organ involvement related to aPL or secondary to other etiologies; the existence of other comorbidities, e.g., smoking, obesity, diabetes; and the risk stratification according to the selected aPL-based risk profile.

### 7.2. Complementary Tests during Pregnancy

In addition to the routine first and second trimester ultrasonography screening, patients with OAPS should undergo supplementary surveillance in the third trimester, at least at monthly intervals, based on biometric and Doppler findings to diagnose early or late PE and/or FGR and plan the time of delivery [22]. In all these pregnant women, a serial blood test should be performed, emphasizing them in those women with a history of placental insufficiency-related complications or with current or past renal involvement. In addition, blood pressure and 24-h urine proteinuria should be regularly monitored. In the next sections, we are going to focus on the drug management of women with OAPS.

### 7.3. General Therapeutic Measures

Folic acid supplementation for at least 1 month prior to conception and throughout pregnancy is recommended. In addition, calcium and vitamin D administration during pregnancy and puerperium should be strongly recommended, particularly in thin pregnant women or when they are taking heparin or prednisone. In cases of immobility, in those with a past or current thrombotic event or even, in general, the use of compression stockings may reduce the thrombotic risk, along with the standard of care [156].

### 7.4. Gold Standard Treatment

Currently, despite limited evidence, the gold standard of treatment is preconception LDA (100 mg/day vo) combined with LMWH at prophylactic doses (0.4–0.6 mg/kg/day; 4000–6000 IU/day by sc.) from the moment of the positive pregnancy test [6]. In patients with previous thrombotic events, secondary thromboprophylaxis with full unfractionated or LMWH doses or anti-vitamin K therapy should be administered [157]. The latter must be avoided between weeks 5 and 12 of gestation (Table 5).

In 1992 Cowchock et al. [158] first published a clinical trial comparing the efficacy of heparin vs. prednisone in the treatment of aPL-associated recurrent pregnancy losses (RPL). Since then, numerous studies have evaluated the effectiveness and safety of heparins in OAPS. In 2020, a meta-analysis by Hamulyák et al. [23] found eleven studies in which the efficacy of heparin was randomized alone or in combination with aspirin to treat patients with RPL associated with aPL positivity. The main conclusion was that heparin alone or associated with LDA was superior to LDA alone or placebo in the treatment of these cases. In contrast, treatment with aspirin alone has no effect on improving the live birth rate. Usually, LDA combined with LMWH treatment is continued throughout pregnancy and 6 weeks postpartum. The mechanism by which heparin can prevent RM/RPL related to aPL seems to go beyond their anticoagulant activity [159]. In brief, and as has been previously commented on, LMWH is able to prevent activation of the complement system [160], which is part of the main pathogenesis of aPL-related endothelial damage [70]. The inhibition of C5a will prevent the release of inflammatory and prothrombotic molecules such as TNF-α and TF [161]. On the other hand, the ability to inhibit the formation of the TF–Factor Xa complex will directly influence the activation of PAR-2 receptors, membrane proteins involved in the mechanism of inflammation and neutrophil infiltration [71]. Thus, it has been reported that LMWH in prophylactic daily doses can prevent 80% of FL and RM [7]. In the puerperium, maintaining LMWH prophylaxis for 6–8 weeks is recommended to prevent blood clot formation in the mother [9]. In cases of documented hypersensitivity to LMWH, the use of fondaparinux is a good option before the allergy tests are performed.

### 7.5. Refractory Cases

Up to 20–30% of cases will not respond to SC. These cases are named refractory OAPS. In this situation, other treatment schedules, mainly HCQ, low-prednisone dose, increasingly LMWH dose, IVIG or plasma exchange, have been tried, with good results [148] (Table 6).

#### 7.5.1. Hydroxychloroquine

HCQ is usually administered to patients with SLE, especially those with recent disease activity, renal involvement or in cases who tested positive for anti-Ro antibodies [162]. In the last years, HCQ has been recommended as a basal treatment to prevent lupus flares.

HCQ improves aAnA5 expression [163], reduces aPL binding to syncytiotrophoblasts [164], shows anti-platelet action [165] and decreases inflammation through its action on TNF-α and exerts a complement pathway inhibition [166]. It has been reported that HCQ is safe during pregnancy and breastfeeding [167]; thus, serious side effects associated with HCQ exposure are scarce; even the EULAR has permitted its use for OAPS treatment [22].

Three retrospective studies from Mekinian et al. [168], Sciascia et al. [169] and Ruffatti et al. [170] showed good results when HCQ was added to the recommended schedule in cases with RPL and high-risk laboratory aPL profiles. Currently, there are three ongoing randomized controlled trials called “HYPATIA” [171], “HIBISCUS” [172] and HYDROSAPL [173] in which the addition of HCQ to SC appears to be beneficial to pregnant women with aPL/OAPS. Recently, Gerde et al. [174] selected 101 pregnancies out of 87 women with OAPS and a previous pregnancy failure despite the conventional treatment. They showed that the addition of HCQ in these patients with primary OAPS was associated with a higher rate of live births and a lower frequency of pregnancy complications.

#### 7.5.2. Corticosteroids

Corticosteroids were the first treatment used in combination with LMWH to treat recurrent pregnancy loss. In 1985, the association of RM and thrombosis with LA was reported, indicating a combined treatment with heparin, aspirin and prednisone in three patients [175]. In 1989, Huang et al. [176] reported two cases of RPL in association with LA that were treated with prednisolone and LDA when successful. Along these lines, Quenby et al. [177] suggested the potential beneficial effects of corticosteroids in women with RM, owing to their ability to reduce the number of NK cells.

Corticosteroids act over diverse immune pathways but, overall, by inhibition of complement activation [178] and producing the apoptosis of T-lymphocytes and granulocytes [179]. In cases of RM/RPL, corticosteroids favor the establishment of early pregnancy by the suppression of dNK cells and improvement of trophoblast proliferation and invasion [180]. Glucocorticoid therapy on cycle days 1–21 in RPL significantly reduced the dNK levels [181]. Recently, Bramham et al. [182] published their results on a cohort of women with refractory OAPS treated with early low-dose prednisolone in addition to LDA and prophylactic heparin who achieved 61% live births. Beyond the fact that high doses of corticosteroids can induce hypertension or diabetes, their use can be considered safe at low doses (≤10 mg/kg/day) due to the few adverse effects observed [183]. In the same way, Palmsten et al. [184] compared the risk of preterm birth in patients with rheumatoid arthritis when treated with prednisone and showed an association between doses ≥10 mg/kg/daily) and preterm births, while doses ≤10 mg/kg daily were not associated with preterm births. At last, focusing on transplacental passage, the metabolism of non-fluorinated glucocorticoids by the placenta ensures that only 10% of inactive metabolites cross the placenta to the fetal circulation at maternal doses of less than 20 mg [185].

#### 7.5.3. Intravenous Immunoglobulins

Intravenous immunoglobulins (IVIG) are being used in some autoimmune-mediated diseases, with varying degrees of success [186]. In RPL, IVIG exert their functions, reducing the presence of autoantibodies, neutralizing them and decreasing NK cell activity. To date, nine trial studies have been registered that seek to demonstrate the efficacy of IVIG in the treatment of RPL, with diverse results [187,188,189,190,191,192,193,194,195]. Currently, however, few data on the usefulness of IVIG in OAPS are available. Urban et al. [196] reported that three patients with OAPS and a history of stillbirths were treated with prophylactic IVIG, in addition to HCQ, LDA, low molecular weight heparin and prednisone, achieving good results, and this was also confirmed in a metanalysis performed by Ruffatti et al. [170]. The same group analyzed high-risk APS, including OAPS patients, and showed that IVIG administration was associated with a significantly higher live birth rate when compared with oral scheduled treatments for refractory cases [197].

#### 7.5.4. Biologic Therapy: TNFα-Targeted Therapies

TNF-α is a cytokine that plays a crucial role in causing inflammation by means of predominantly T-cell-mediated tissue damage [198]. The four most commonly anti-TNF-α drugs used are infliximab, etanercept, adalimumab and certolizumab. Infliximab is a chimeric monoclonal antibody against TNF-α administered as an iv infusion [199]. Etanercept, a p75 TNF–receptor fusion protein conjugated to the Fc region of human IgG immunoglobulin, is administered as a sc injection twice weekly [200]. Adalimumab is a human anti-TNF-α monoclonal antibody also administered sc every two weeks [201]. Certolizumab pegol is a Fc-free PEGylated TNF-α blocker approved for the treatment of psoriatic arthritis and plaque psoriasis in many countries [202]. All these drugs were tested involuntarily in women with inflammatory diseases who became pregnant whilst under TNF-targeted treatment, no significant fetal adverse effects were observed [203].

Alijotas-Reig et al. [60] showed how the treatment with TNF-α blockers in 18 patients with refractory aPL/OAPS was accompanied by good obstetric results: 70% were successful, without adverse maternal–fetal effects. To date, only one study has been registered in the search of the efficacy of TNF-α blockers in the treatment of RPL in OAPS patients: the IMPACT study determined whether TNF-α blockade during pregnancy, added to a standard of care, could reduce obstetric morbidity, decreasing inflammation and improving poor placental vascularization. The TNF-alpha inhibitor selected was Certolizumab, a TNF-α inhibitor that does not cross the placenta (IMPACT Study: IMProve Pregnancy in APS with Certolizumab Therapy, NCT03152058).

### 7.6. Other Drugs Tested in the Treatment of OAPS

Agmon-Levin et al. [204] found low vitamin D levels in APS patients and associated them with thrombotic complaints. These authors also showed, in vitro, that vitamin D exert its antithrombotic effect via inhibition of the endothelial cell expression of TF. Fortunately, as previously commented, vitamin D supplementation is currently recommended in OAPS prepregnant and pregnant women. In the same way, the use of statins, which have pleiotropic anti-inflammatory and immunomodulating properties, has shown good results. Statins do not exert adverse effects on embryos and fetuses in animals and humans [205]. The combination of LMWH, LDA and pravastatin significantly improved placental hemodynamics and maternal and neonatal outcomes in women and mice with OAPS [57,58]. Eculizumab inhibits terminal complement activation and is apparently effective and safe in the treatment of thrombotic microangiopathy (CAPS) and in the prevention of thrombotic events during pregnancy, both in the mother and newborn. Eculizumab could be a tool to be used in selected cases of refractory OAPS [206,207]. Finally, and has been previously commented, rituximab has been used in CAPS induced by or complicated pregnancy with success and no adverse effects.

### 7.7. Antithrombotic Drug Management during Delivery and Early Puerperium

Another important aspect is the management of antithrombotic and antiaggregant drugs in these women during delivery and the puerperium period. The fear to may provoke hemorrhagic complications, particularly in relationship with neuroaxial procedures has been a constant over time. To avoid or minimize this bleeding risk, discontinuation of LDA has been suggested and it depends mostly of hospital protocols and anesthesiologist experience, but we recommend to do not stop until the delivery day in cases of women diagnosed with OAPS. In addition, it is recommended that OAPS patients on anticoagulation with LMWH may follow until the end of gestation or at week 36–37 it could switch to unfractionated heparin and stop 4–6 h prior to elective induction of delivery, caesarean section, or neuroaxial anesthesia [208] However, if the patient is maintained on prophylactic/therapeutic LMWH, this should be suspended 12–24 h, respectively prior to elective induction of delivery, caesarean section, or neuroaxial anesthesia. Recently, Alijotas-Reig et al. [209], after analyzing a large cohort of OAPS patients treated with SC until the end of pregnancy showed no increase in major bleeding in these patients, thus reassuring obstetricians and anesthesiologists in this regard.

## 8. Conclusions

Obstetric antiphospholipid syndrome (OAPS) is currently an accepted subset of APS. OAPS diagnosis requires the absence of previous thrombotic events. Venous and/or arterial thrombosis, although less frequently than in APS, may complicate the follow-up of these patients. The mechanism of action of OAPS appears to be primarily related to the activation of the proinflammatory pathway, mainly throughout complement proteins and TNF-α, driven by the interaction of phospholipid–aPL complexes on trophoblast, platelets and innate immune cells with the further delivery of antiangiogenic molecules and TF. Interestingly, only 50% of placentas of OAPS women showed signs of thrombosis. Overall, recurrent pregnancy losses are the mainstay of clinical manifestations, followed by those related to early placental insufficiency. Triple positivity is the laboratory category most related to poor obstetric outcomes. OMAPS and NC-OAPS cases are still challenging for clinicians. A better categorization of these patients is crucial to not underdiagnose them and to avoid possible further pregnancy complications, as well as thrombotic events. New laboratory technologies aiming to better detect aPL are currently under evaluation. In addition, the analysis of so-called non-criteria aPL could improve the management of NC-OAPS. A redefinition of the Sydney criteria or the construction of new diagnostic tools will help physicians in this field. The SC is based on the combination of LDA and prophylactic LMWH. In refractory cases, therapy with HCQ and/or low-dose prednisone is recommended. High-risk and recurrent refractory cases could receive therapies based on TNF-α inhibitors, IVIGs, plasma exchange and complement inhibitors. All of these therapeutic agents have been tried, with preliminary good results.

## Figures and Tables

**Table 1:** Clinical and laboratory descriptions for OAPS and its variants OMAPS and NC-OAPS.

**OAPS**	**Clinical Criteria**
	1. ≥3 consecutive miscarriages before week 10 of gestation
	2. At least one fetal loss after week 10 of gestation
	3. At least one premature birth before week 34 of gestation due to PE/eclampsia or placental insufficiency
	**Laboratory criteria**
	1. Two LA positive tests at least 12 weeks apart
	2. Two IgG or IgM aCL positive tests at least 12 weeks apart
	3. Two IgG or IgM aβ2GPI positive tests at least 12 weeks apart
**OMAPS**	**Clinical criteria**
	1. Two consecutive unexplained miscarriages of well-formed embryos
	2. Three or more non-consecutive miscarriages of well-formed embryos
	3. PE/eclampsia after week 34 of gestation or at puerperium
	4. Placental abruption
	5. Late premature birth
	6. Premature rupture of membranes
	7. Unexplained recurrent implantation failure in in vitro fertilization *
	**Laboratory criteria**
	Fulfil the laboratory criteria described for OAPS
**NC-OAPS**	**Clinical criteria**
	Fulfil the clinical criteria described for OAPS
	**Laboratory criteria**
	1. Positivity for LA, aCL or aβ2GPI only detected once
	2. Low positive IgG/IgM aCL or aβ2GPI titers
	3. Persistent positivity for non-criteria aPL, including IgA-aCL and aβ2GPI
	4. Resistance to Annexin A5 anticoagulant activity
	5. Thrombocytopenia

* Failure of at least 3 embryo transfer of good-quality embryos. aCL: IgG/IgM anticardiolipin antibodies; aβ2GPI: IgG/IgM antiβ2-glycoprotein I antibodies; aPL: antiphospholipid antibodies; LA: lupus anticoagulant; OAPS: obstetric antiphospholipid syndrome; NC-OAPS: non-criteria OAPS; OMAPS: obstetric morbidity antiphospholipid syndrome; PE: pre-eclampsia.

**Table 2:** Histopathological findings suggestive of the OAPS.

Tissue Affectation	Histologic Features aPL-Related	References	Histologic Features aPL Non-Related	References
Decidua	Necrosis	[32,33]		
	Acute Inflammation	[32]		
	Chronic Inflammation	[32]		
	Partial Remodeling spiral arteries	[34]	Complete remodeling spiral arteries	[32,34]
	Vessel thrombus	[32,35]		
Placenta	Villous infarcts	[27,32]	Placental infarction	[25]
	Hydropic villi	[32]	Intervillous fibrin	[32]
	Stromal fibrosis	[27,32]	Hemorrhagic endovasculitis	[32]
	Syncytial knots	[32]	Syncytial knots	[34]
	Complement deposition C4d	[36,37]	Complement deposition C3	[38]
	Intervillous thrombus	[27,32,35]	Intervillous thrombus	[25]

**Table 3:** Variations of the antiphospholipid antibody titers during pregnancy.

Treatment	aPL Titers		Refs.
	Fluctuated	Cleared	
prednisone plus LDA	Yes	No	[115]
LMWH ± LDA	Yes	Yes	[114]
no treatment *	Yes	Yes	[112]
unfractionated heparin	Yes	Yes	[116]
LMWH plus LDA	Yes	No	[119]
LMWH	Yes	No	[113]
LMWH	Yes	Yes	[118]
LMWH	aPL-PL reduction binding		[118]
LMWH	aPL-PL reduction binding		[117]

* Healthy pregnant women. aPL: antiphospholipid antibodies; LDA: low-dose aspirin; LMWH: low molecular weight heparin; OAPS: obstetric antiphospholipid syndrome.

**Table 4:** The Global Antiphospholipid Syndrome Score (GAPSS) and the adjusted GAPSS (aGAPSS).

Feature	GAPSS	aGAPSS
aCL	5	5
aβ2GPI	4	4
LA	4	4
aPS/PT complex	3	-
Hyperlipidemia	3	3
Arterial hypertension	1	1
Total	20	17

**Table 5:** Suggested therapeutic schedules for OAPS patients.

Gold Standard therapy in spontaneous pregnancy loss: recurrent miscarriage/fetal loss	LMWH 0.4–06 mg/kg/day (“prophylactic” dose) since the positive pregnancy test combined with preconception daily LDA at least one month before starting attempts for a new pregnancy.
Gold Standard Therapy in assisted reproductive techniques (ART)	LMWH 0.4–0.6 mg/kg/day since estrogens are started in the substituted cycle (or 14 days prior to the transfer, if not), combined with preconception LDA, at least one month before starting ART
Women with a previous history of thrombotic APS or thrombosis that appeared during pregnancy	LMWH 1 mg/kg/12 h since the thrombotic event, combined with LDA.
Presence of severe thrombocytopenia (less than 20,000 platelets) or presence of mild-moderate bleeding	Stop LDA LMWH 0.2 mg/kg/day in the case of OAPS LMWH 1 mg/kg/day in thrombotic APS Monitor total platelet count Monitor anti-factor Xa activity
Presence of mild-moderate renal failure (GFR 15–45 mL/min)	Reduce the LMWH dose that was administered and discontinue aspirin. Monitor anti-factor Xa activity monthly
Presence of extreme weights (less than 40 kg or greater than 120 Kg)	LMWH 0.2 to 0.8 mg/kg/day (prophylactic dose adjusted to body weight), since positive pregnancy test combined with preconception LD., Monitoring anti-factor Xa activity monthly.

APS: antiphospholipid syndrome; ART: assisted reproductive techniques; GFR: glomerular filtration rate; LDA: low-dose aspirin; LMWH: low molecular weight heparin; OAPS: obstetric antiphospholipid syndrome.

**Table 6:** Recommended steps in the treatment of refractory OAPS.

Step 1	ASA 100 mg started 4 weeks before gestation. Prophylactic LMWH from the time of knowing pregnancy
Step 2	Step 1 + HCQ 200–400 mg/day, according to body weight
Step 3	Step 2 + low-dose prednisone 10 to 15 mg/d (started as soon as pregnancy is known) or increasing LMWH dose.
Step 4	Step 3 + add IVIGs and/or perform plasma exchange
Step 5	Step 4 + add anti-TNF (infliximab, etanercept, but better use adalimumab or certolizumab).
Step 6	Consider adding hydrophilic statins (pravastatin 20–40 mg/day), or eculizumab.

Updated from Reference [148]. ASA: acetylsalicylic acid; HCQ: Hydroxychloroquine; IVIGs: intravenous immunoglobulins; LMWH: low molecular weight; OAPS: obstetric antiphospholipid syndrome; RPL: recurrent pregnancy loss (includes recurrent miscarriage, recurrent fetal loss and stillbirths); TNF: tumor necrosis factor.

## Data Availability

Not applicable.
